# Retraction: Synthesis and characterization of AFe_2_O_4_ (A: Ni, Co, Mg)–silica nanocomposites and their application for the removal of dibenzothiophene (DBT) by an adsorption process: kinetics, isotherms and experimental design

**DOI:** 10.1039/d3ra90069j

**Published:** 2023-07-31

**Authors:** Fahimeh Vafaee, Samira Mandizadeh, Omid Amiri, Mansour Jahangiri, Masoud Salavati-Niasari

**Affiliations:** a Faculty of Chemical, Petroleum and Gas Eng., Semnan University P. O. Box, 35196-45399 Semnan Islamic Republic of Iran mjahangiri@semnan.ac.ir +98 31 55913201 +98 31 55912383; b Institute of Nano Science and Nano Technology, University of Kashan P. O. Box 87317-51167 Kashan Islamic Republic of Iran salavati@kashanu.ac.ir; c Faculty of Chemistry, Razi University 6714414971 Kermanshah Iran; d Department of Chemistry, College of Science, University of Raparin Rania Kurdistan Region Iraq

## Abstract

Retraction of ‘Synthesis and characterization of AFe_2_O_4_ (A: Ni, Co, Mg)–silica nanocomposites and their application for the removal of dibenzothiophene (DBT) by an adsorption process: kinetics, isotherms and experimental design’ by F. Vafaee *et al.*, *RSC Adv.*, 2021, **11**, 22661–22676, https://doi.org/10.1039/D1RA02780H.

The Royal Society of Chemistry, with the agreement of the authors, hereby wholly retracts this *RSC Advances* article due to concerns with the reliability of the data.

Following the publication of a previous correction to replace the XRD patterns in Fig 4a and b,^1^ a new concern has been identified with the published article. The XRD patterns in Fig. 2b and both the original Fig. 4a and b contain multiple overlapping sections. In addition, new information brought to the attention of the Editor undermines the integrity of the authors' original explanation, meaning that the Editor has lost confidence that the findings presented in this paper are reliable.

Omid Amiri, Mansour Jahangiri, and Masoud Salavati-Niasari wish to state that they had no role in preparing the XRD data.

This retraction supersedes the information provided in the correction^1^ related to this article.

Signed: Fahimeh Vafaee, Samira Mandizadeh, Omid Amiri, Mansour Jahangiri, Masoud Salavati-Niasari

Date: 24/7/2023

Retraction endorsed by Laura Fisher, Executive Editor, *RSC Advances*

## Supplementary Material
